# Evaluating intersectional variation of HPV-associated cancers in rural America

**DOI:** 10.1186/s12889-025-23963-y

**Published:** 2025-08-02

**Authors:** Jason Semprini, Gabriel Benavidez, Whitney E. Zahnd, Heather M. Brandt

**Affiliations:** 1 Department of Public Health, Des Moines University College of Health Sciences, 8025 Grand Ave, West Des Moines, IA 50266 USA; 2 Robbins College of Health and Human Sciences, Department of Public Health, Baylor University, Waco, TX USA; 3 College of Public Health, Department of Health Management and Policy, University of Iowa, Iowa City, IA USA; 4 St. Jude Children’s Research Hospital, HPV Cancer Prevention Program, Memphis, TN USA

**Keywords:** HPV, Cancer, Equity, Disparities, Rural, Race/ethnicity, Poverty, Sex

## Abstract

**Purpose:**

For decades, incidence of human papillomavirus (HPV)-associated cancers has been increasing in rural communities across the United States. Although emerging evidence shows a widening rural-urban disparity, rural intersectionality has been understudied. Our study examined the incidence of HPV-associated cancers within rural communities to identify differences by race/ethnicity for males and females, and explore how these differences varied by cancer type, socioeconomic, and geographic factors.

**Methods:**

We accessed age-adjusted cancer incidence rates (2010–2019) from the North American Association of Central Cancer Registries (NAACCR) for HPV-associated cancers (cervical, vaginal, vulvar, penile, anal, oropharyngeal) in rural counties. Stratifying by sex, we calculated incidence rate ratios by race/ethnicity. Subgroup analyses included age, site, census-tract poverty, census tract socioeconomics, and region.

**Results:**

Between 2010 and 2019, rural HPV-associated cancer was 11.8 cases per 100,000 population. We found significant heterogeneity within male (10.5) and female (13.2) rates. For males, the lowest rate was found in non-Hispanic Asian-American/Pacific-Islander populations (3.7) and Hispanic populations (4.8), and the highest rate was found in non-Hispanic White populations (11.2). For females, the lowest rate was also found in Hispanic Asian-American/Pacific-Islander populations (8.8) and the highest rates were found in non-Hispanic Black (13.8) and non-Hispanic American Indian/Alaska Native populations (14.5). However, these racial/ethnic differences varied across rural subpopulations, geography, and poverty.

**Conclusions:**

Appreciating the diversity of the rural cancer burden can be used to effectively develop and implement public health interventions to address HPV-related cancer disparities in rural communities. Actions are needed to prioritize reducing the burden of HPV-associated cancer in AIAN populations in high-poverty rural communities.

**Supplementary Information:**

The online version contains supplementary material available at 10.1186/s12889-025-23963-y.

## Introduction

Human papillomavirus (HPV) is the most common sexually transmitted infection in the United States, with approximately 40% of individuals aged 15–59 currently infected with HPV [1]. Twelve oncogenic strains of HPV have been linked to HPV-associated cancer [1]. The estimated lifetime probability of HPV infection is over 80% for both men and women by age 45 [2]. Although the estimated lifetime risk of HPV infection by strain is not well understood, disease-associated HPV infection was 20% in females and 24% in males ([1]– [2]). This is of significant concern given that HPV is the causative agent of most anal, head and neck, oral, oropharyngeal, penile, vaginal, vulvar, and cervical cancers with approximately 40,000 incident cases annually attributed to HPV infections in the United States (U.S.) [3]. While many people in the U.S. are at risk of developing HPV-associated cancers due to the high incidence and prevalence of HPV infections, the advent of the HPV vaccine released in 2006 for females and 2009 for males has resulted in significant declines overtime in the incidence of HPV-associated cancers. In 2001, HPV-associated cervical cancer incidence among 20-24-year-old females was 1.37 cases per 100,000 population [4]. Between 2001 and 2017, cervical cancer incidence declined 5.1% annually. The same nationally representative study found that the incidence in females aged 25–29 and 30–34, significantly declined since the 2001 rates of 6.79 and 12.26, respectively [4].

Unfortunately, decreasing incidence in HPV-associated cancers have not been experienced uniformly across the U.S. population. Multiple studies have found that, rural populations have seen a significant increase in the incidence of HPV-associated cancers over the last two decades [5–7]. In 2000, the incidence rate of all HPV-associated cancers combined were similar between metropolitan urban (11.0 cases per 100,000) and non-metro rural (11.2 cases per 100,000) populations [8]. Then, between 2002 and 2019, rural areas experienced a significant sustained increase in HPV-associated cancer with an average annual percentage change of 1.6% [5]. Similar findings have also been noted when only examining cervical cancer incidence with rural areas demonstrating higher rates of cervical cancer compared to urban areas [9]. The disparities in HPV-associated cancer incidence are largely attributable to disparities in HPV vaccination coverage and cervical screening specifically for cervical cancer. A landmark nationwide study of Swedish women showed that compared to women who were not vaccinated against HPV, those fully vaccinated had approximately 88% lower risk of developing cervical cancer [10]. For a multitude of reasons encompassing limited availability of and access to high-quality primary care, missed opportunities for recommendations and education, and challenges related to cultural differences, HPV vaccination coverage in rural communities is 10–15% lower than among urban counterparts [11–18].

The identification of rural-urban disparities is a necessary first step for advancing equity, as it provides evidence of the unequal cancer burden—specifically the HPV-associated cancer burden experienced in rural communities. Reversing the trend of widening rural-urban disparities in HPV-associated cancer incidence will require all stakeholders to develop and implement targeted interventions to address the unique needs of diverse rural subpopulations. Contrary to popular beliefs, rural America is not and has never been a monolith [19]. Over 24% of all rural residents are members of Hispanic, non-Hispanic Black, Indigenous, Asian racial/ethnic group [20]. In fact, rural America has become increasingly more racially and ethnically diverse over the last two decades [20]. Despite shifting demographic trends, many rural communities still experience persistent poverty and the intersection of rurality, race, and poverty has been identified as a source of elevated cancer mortality [21]. For too long, the research informing interventions to address the unmet needs of rural subpopulations has lagged behind changing demographic trends and disparate disease burdens [22, 23].

In this study, we aimed to investigate disparities, not between, but within rural populations – specifically examining intersectional variations in the rural HPV-associated cancer burden by sex and race/ethnicity. While HPV-associated cancer rates have risen in rural U.S. communities, current evidence largely highlights rural-urban differences [13]. Although rural-urban comparisons can highlight disparities and potential opportunities for intervention, ignoring the variation within an increasingly diversifying rural America risks missing specific groups with elevated cancer burdens. Given the unique features of HPV-associated cancer between females and males, the growing racial/ethnic diversity within rural US, and the place-based differences in poverty and health systems, our findings can improve our comprehensive understanding of rural HPV-associated cancer disparities and provide insight for cancer control planners and implementers to employ precise, rural-specific interventions tailored to the diverse needs within rural populations.

## Methods

### Data

We used data from the North American Association of Central Cancer Registries (NAACCR), a collaborative organization of state, province, and central registries across the U.S. and Canada [24]. With the goal of providing uniform standards for collecting and reporting cancer data across the continent, NAACCR provides the most comprehensive population-based cancer registry data for all 50 states and the District of Columbia (DC). For this study, we accessed NAACCR data via the statistical program “SEER*Stat”. NAACCR suppresses all incidence rate calculations if the selection results in fewer than 16 cases.

### Inclusion criteria

Our inclusion criteria were HPV-associated cancers in rural, U.S. counties diagnosed between 2010 and 2019. First, we used the Center for Disease Control and Prevention (CDC) definitions and predefined dictionary to select only cancers associated with HPV as defined by site, histology, and microscopically confirmed diagnoses [25]. These cancers included specific oropharynx, anal and rectal, vulvar, vaginal, cervical, and penile cancers. See supplemental exhibit 1a for full case selection with site-specific ICD-codes [25].

NAACCR also provides 2013 Rural Urban Continuum Codes (RUCC), which categorizes counties based upon their population size and level of urbanization [26]. Here, rural was defined as a RUCC 4–9, or any county not classified as metropolitan [27]. We excluded all metropolitan rates and rates with missing or unknown metropolitan status. Our analysis only included rural rates in the 50 states and DC. We pooled the incidence over this ten-year period to overcome data suppression limitations in small populations and to avoid issues with inferring differences during the initial years of the COVID-19 pandemic [28, 29].

### Stratifying factors

Given the differences in HPV-associated cancer risk, we stratified all analyses by male and female [30]. Within each sex, we further stratified to report different rates for each of the following groups based on self-reports to the diagnosing medical facility: non-Hispanic White; non-Hispanic Black; Hispanic, non-Hispanic Asian/Pacific Islander; non-Hispanic American Indian/Alaska Native [31]. We did not analyze rates for populations with an unknown race/ethnicity. Note, NAACCR only includes American Indian/Alaska Native rates for populations residing in a Purchased/Referred Care Delivery (PRCD) Area [32].

For each sex-stratified, we also reported rates for each race/ethnicity across the following subgroups: age, cancer site, census-tract poverty-level, census tract socioeconomic status, and geographic region. For age, we categorized subgroups by intervals (0–29, 30–49, 50–64, 65–79, 80+). For cancer site in males, we separately analyzed oropharynx cancers, and all “other” sites combined. For females, we separately analyzed cervix cancers, oropharynx cancers, and all other sites combined. For census-tract poverty-level, we used NAACCR’s derived variable which stratifies census-tract by the proportion of the census-tract living in poverty (< 10%, 10–20%, > 20%). NAACCR also provides a census tract socioeconomic measure called the Yost Quintile [33]. Created from a composite index of socioeconomic indicators, the Yost Quintiles offer a within-state metric ranking low [1] to high [5] socioeconomic status at a granular level. We reported incidence rates for each of the five quintiles.

Finally, we analyzed sex-specific incidence rates for each racial/ethnic population for four different geographic regions and geographic regions by census-tract poverty level. The regions closely follow US census regions, but were adjusted to account for cultural context and grouped as follows: We defined these regions as: Northeast (Connecticut, Delaware, Maine, Maryland, Massachusetts, New Hampshire, New Jersey, New York, Pennsylvania, Rhode Island, Vermont, Washington, D.C.); Midwest (Illinois, Indiana, Iowa, Kansas, Michigan, Minnesota, Missouri, Nebraska, North Dakota, Ohio, South Dakota, Wisconsin); Southeast (Alabama, Arkansas, Florida, Georgia, Kentucky, Louisiana, Mississippi, North Carolina, Oklahoma, South Carolina, Tennessee, Texas, Virginia, West Virginia); West (Alaska, Arizona, California, Colorado, Hawaii, Idaho, Montana, Nevada, New Mexico, Oregon, Utah, Washington, Wyoming).

### Statistical analysis

All incidence rates were age-adjusted using the 2000 standard population and reported per 100,000 population with corresponding 95% confidence intervals (CI) of each rate, using the Tiwari modification method [34]. Cancer case and population counts and populations were also reported. To determine if incidence rates differed statistically significantly across racial/ethnic groups, we calculated Incidence Rate Ratios (IRR). Using the non-Hispanic White population as the reference group, we reported IRRs with corresponding CIs and p-values. Given that each calculation involves four tests, we used the Bonferroni method to set our level of statistical significance at alpha = 0.0125 [35].

## Results

### Summary statistics

Between 2010 and 2019, there were 65,828 HPV-associated cancers diagnosed in residents (with known race/ethnicity) of rural U.S. counties (Supplemental Exhibit 2). An additional 494 HPV-associated cancers were diagnosed in rural counties without known race/ethnicity data, comprising just 0.7% of all diagnoses (Supplemental Exhibit 1b). Table 1 presents HPV-associated cancer case counts in rural U.S. counties from 2010 to 2019 by sex, race/ethnicity, age, site, poverty level, and region. Most cases occurred in non-Hispanic White (NHW) males (28,496) and females (29,404). The second largest racial/ethnic group by counts were found in non-Hispanic Black individuals, with higher cases in in counties with > 20% poverty and the southeast.

**Table 1:** Summary Statistics – Case counts of HPV-associated Cancer (2010–2019)

**Overall**	Male					Female				
	NHW	NHB	Hispanic	API	AIAN	NHW	NHB	Hispanic	API	AIAN
	28,496	1,826	625	88	325	29,404	2,665	1,593	266	540
Age										
0–29	*< 16*					761	81	93	*< 16*	39
30–49	2830	278	88	*< 16*	47	7936	800	764	120	203
50–64	14,561	983	312	35	177	11,302	1004	469	88	188
65–79	9598	509	192	34	95	7377	590	227	38	88
80+	1484	48	33	*< 16*	*< 16*	2028	190	40	16	22
Site										
Oropharyngeal	24,448	1,498	488	83	277	5,409	359	100	16	55
Cervix	n/a					15,265	1,855	1,273	216	408
All Other Sites	4048	328	137	< 16	48	8730	451	220	34	77
Poverty										
< 10%	6281	75	92	21	34	5731	90	169	74	40
10–20%	13,051	472	224	42	141	13,425	679	617	107	182
> 20%	7477	1151	275	20	133	8584	1733	697	74	277
Region										
Northeast	3514	47	28	*< 16*	*< 16*	3292	44	52	*< 16*	*< 16*
Midwest	9637	101	87	*< 16*	74	10,116	124	234	54	124
Southeast	11,573	1654	312	23	117	12,431	2478	879	77	177
West	3772	24	198	47	119	3565	19	458	121	230

Cells with less than 16 cancer cases were suppressed

*API* Asian/Pacific Islander, *AIAN* American Indian/Alaska Native

### Primary results

The overall age-adjusted incidence rate was 11.8 cases per 100,000 population. Incidence was lower in males (Rate = 10.5; C.I. = 10.4, 10.6) than females (Rate = 13.2; C.I. = 13.1, 13.4) (Supplemental Exhibit 2). Within males, the lowest age-adjusted incidence rate of HPV-associated cancer was found in non-Hispanic Asian/Pacific Islander populations (Rate = 3.7, C.I. = 3.0, 4.6) and Hispanic populations (Rate = 4.8) (Table 2). In males, the highest overall age-adjusted incidence rate of HPV-associated cancer was found in non-Hispanic White populations (Rate = 11.0; C.I. = 10.9, 11.1). The male non-Hispanic White population incidence rate was significantly higher than the male incidence rate in every other racial/ethnic group.

**Table 2:** HPV-Associated Cancer incidence rates (2010–2019), by sex and race/ethnicity and subgroups

**Overall**	Male					Female				
	NHW	NHB	Hispanic	API	AIAN	NHW	NHB	Hispanic	API	AIAN
	11.0	9.3#	4.8#	3.7#	9.2#	13.3	13.8	11.8#	8.8#	14.5
Age										
0–29	*Suppressed*					1.2	1.0	1.1	*Suppressed*	2.0#
30–49	6.0	5.4	1.8#	*Suppressed*	5.0	18.7	18.0	17.6	14.3#	20.8
50–64	34.2	27.4#	13.1#	8.0#	27.5#	27.2	27.6	22.3#	16.5#	26.9
65–79	35.8	32.3#	19.9#	17.6#	34.7	25.9	30.8#	23.3	13.1#	26.4
80+	20.7	14.6#	14.0#	*Suppressed*	*Suppressed*	18.1	27.1#	12.9#	17.5	23.8
Site										
Oropharyngeal	9.4	7.4#	3.7#	3.5#	7.8#	2.0	1.7#	0.8#	0.5#	1.4#
Cervix	n/a					8.0	10.0#	9.2#	7.1	11.2#
All Other Sites	1.7	1.8	1.1#	*Suppressed*	1.5	3.3	2.2#	1.8#	1.1#	2.0#
Poverty										
< 10%	2.4	0.4#	0.7#	0.8#	1.0#	2.5	0.5#	1.2#	2.5	1.1#
10–20%	5.0	2.4#	1.7#	1.8#	4.0#	6.1	3.5#	4.6#	3.5#	4.8#
> 20%	2.9	5.8#	2.1#	0.8#	3.8#	4.0	9.0#	5.2#	2.5#	7.5#
Region										
Northeast	10.9	7.5#	7.4	*Suppressed*	*Suppressed*	11.7	11.7	11.9	*Suppressed*	*Suppressed*
Midwest	10.2	6.8#	4.4#	*Suppressed*	11.2	12.6	12.8	10.8#	8.8#	17.6#
Southeast	12.1	9.7#	4.8#	3.9#	12.5	15.0	14.0#	13.2#	9.8#	17.9#
West	10.2	5.6#	4.7#	3.7#	6.4#	12.0	8.7	10.2#	8.9#	11.9

*API* Asian/Pacific Islander, *AIAN* American Indian/Alaska Native

^#^The rate ratio indicates that the rate is significantly different than the rate for Non-Hispanic White (p<0.0125)

We observed less statistically significant variation across female rates. The lowest incidence of HPV-associated cancer was found in non-Hispanic Asian/Pacific Islander populations (Rate = 8.8; C.I. = 7.7, 9.9), which was lower than every other racial/ethnic group. In females, the incidence rate in the Hispanic population was also lower than the remaining three racial/ethnic groups (Rate = 11.8; C.I. = 11.2, 12.4). While the highest observed incidence rate was found in the non-Hispanic American Indian/Alaska Native population (Rate = 14.5; C.I. = 13.3, 15.8), there was no statistically significant differences between this rate and the rates in the non-Hispanic Black or non-Hispanic White populations.

### Age

In males under 30 years old, all cancer data were suppressed due to low counts, whereas female rates ranged from 1.0 in non-Hispanic Black populations to 2.0 in AIAN populations. (Table 2). Across every age group over 50-years in males, incidence of HPV-associated cancer was highest in non-Hispanic White populations. The highest incidence in males were found in 65–79-year-old populations (NHW = 35.8, AIAN = 34.7) Among females, for ages 30–49, 50–64, and 65–79, the lowest rates of HPV-associated cancer were found in API. There were few differences across other racial/ethnic groups in younger ages. In the 65–79 and 80 + age groups, however, the HPV-associated cancer incidence of 30.8 and 27.1, respectively, in non-Hispanic Black females was significantly higher than other racial/ethnic groups.

### Site

Rural HPV-associated oropharyngeal cancer incidence was higher in males than females across all racial/ethnic groups. The highest male incidence of HPV-associated oropharyngeal cancer was found in non-Hispanic White populations (9.4) and lowest in Hispanic (3.7) and API populations (3.5). In females, the rate of oropharyngeal cancer was higher in non-Hispanic White populations (2.0) than all other racial/ethnic groups. Cervical cancer incidence was lowest in non-Hispanic White (8.0) and API populations (7.1). The rate of cervical cancer in Hispanic populations (9.0) was significantly higher than the rate in non-Hispanic White populations. The highest incidence of cervical cancer was found in non-Hispanic Black (10.0) and AIAN populations (11.2).

### Census-tract poverty-level

In rural census-tracts with < 10% of the population living in poverty, HPV-associated cancer incidence was significantly higher in non-Hispanic White males (2.4) than all other male racial/ethnic groups, and non-Hispanic White (2.5) and API females (2.5) than all other female racial/ethnic groups. In these census-tracts with < 10% of the population living in poverty, the lowest incidence of HPV-associated cancer was found in non-Hispanic Black males (0.4) and non-Hispanic Black females (0.5). Although incidence rates were higher across all groups, in census-tracts with 10–20% of the population living in poverty, HPV-associated cancer incidence was also higher in non-Hispanic White males (5.0) and non-Hispanic White females (6.1) than all other racial/ethnic groups.

In rural census-tracts with > 20% of the population living in poverty, the incidence of HPV-associated cancer in males was highest in non-Hispanic Black populations (5.8), which was significantly higher than all other groups. In rural census-tracts with > 20% of the population living in poverty, the female incidence of HPV-associated cancer was highest in non-Hispanic Black (9.0) and AIAN (7.5). For both male and female rates in these rural census-tracts with > 20% of the population living in poverty, the lowest rates were found in API populations.

### Geographic regions

In the rural Northeast region, the lowest incidence of HPV-associated cancer in males was found in non-Hispanic Black populations (7.5). There was no statistically significant variation across racial/ethnic groups in females in the northeast. In the rural Midwest, male incidence was lowest in non-Hispanic Black (6.8) and Hispanic populations (4.4) and in females the incidence was lowest in API populations (8.8). The female incidence in rural Midwest AIAN populations (17.6) was higher than all other female racial/ethnic groups in the region. In the Southeast, the lowest male incidence was found in Hispanic (4.4) and API populations (3.9), and for females the lowest rate was found in API populations (9.8). Again, the female incidence in AIAN populations (17.9) was higher than all other groups in the region. Finally, in the West, male incidence in the non-Hispanic White population (10.2) was higher than all other groups, and the rates in females were highest in non-Hispanic White (12.0) and AIAN populations (11.9 per 100,00).

**Fig. 1:**
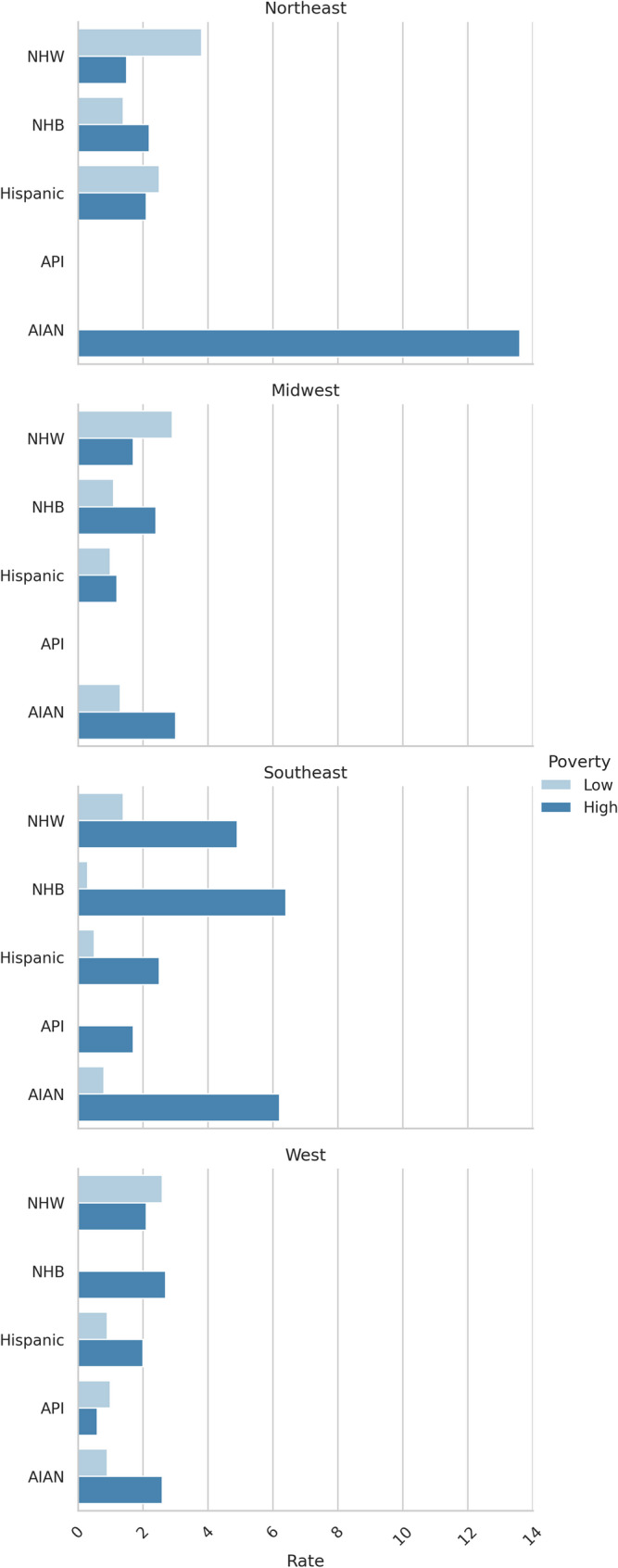
Male HPV-associated cancer incidence by race/ethnicity, region, and census-tract -level poverty

**Fig. 2:**
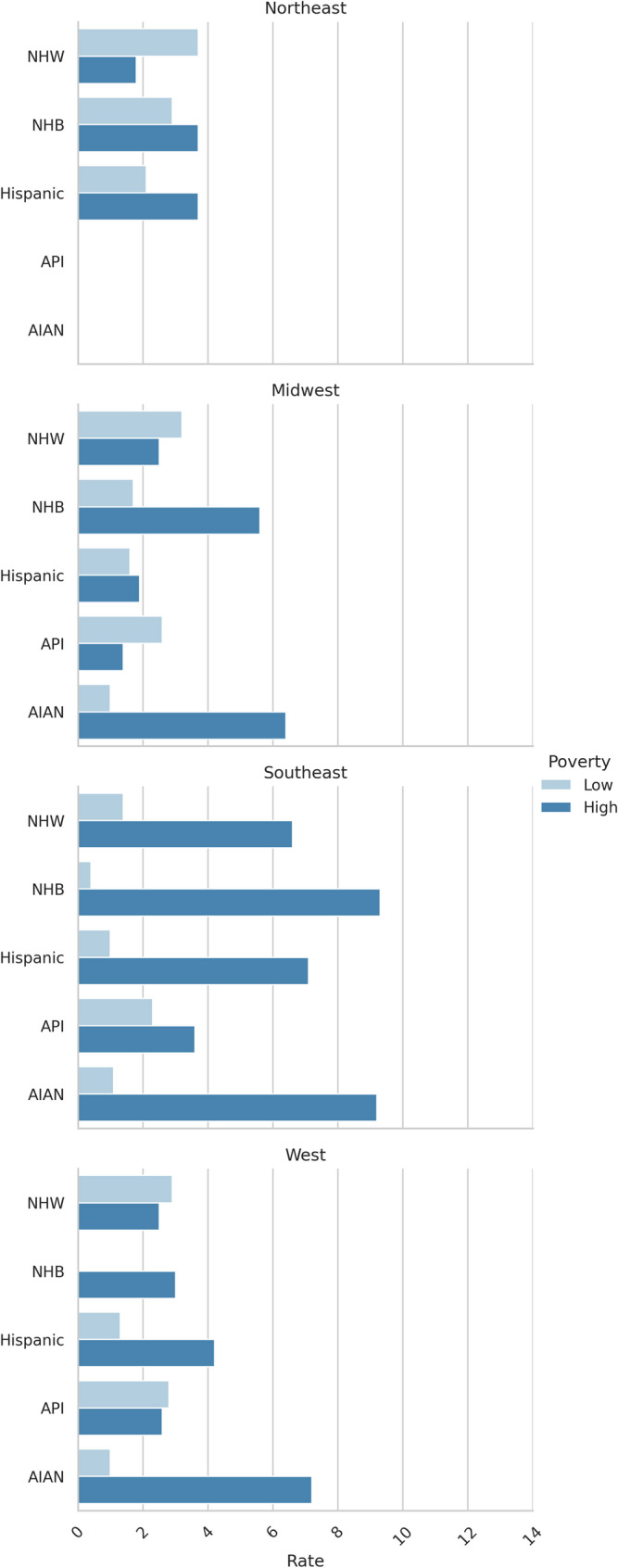
Female HPV-associated cancer incidence by race/ethnicity, region, and census-tract -level poverty

Figures 1 and 2 show the incidence of rural HPV-associated cancers for males and females, stratified by race/ethnicity, geography, and census-tract -level poverty measures.

Figure 1 stratifies age-adjusted HPV-associated cancer incidence rates (per 100,000 population) in males by race/ethnicity, geographic region, and census-tract -level poverty. Low poverty = <10% of census-tract lives in poverty. High poverty = >20% of census-tract lives in poverty. See supplemental exhibit 1a for regional classification for each state. Missing rates indicate data was suppressed due to low counts (< 16 cases).

## Discussion

Using an intersectional approach, the findings of this study underscore the significant and persistent disparities in HPV-associated cancer incidence within rural populations in the United States. We found considerable heterogeneity within and across racial/ethnic groups in both male and female incidence of HPV-associated cancer in rural America. These patterns varied by sex and race/ethnicity differently across geography and poverty levels. Among males, incidence was generally highest in non-Hispanic White populations, but in some geographic regions AIAN and non-Hispanic Black males residing in high-poverty areas had some of the highest rates. Among females, incidence was relatively more consistent across racial/ethnic groups, but was notably higher in AIAN and non-Hispanic Black women residing in high-poverty areas of the Midwest and Southeast. These differences suggest that a “one size fits all” approach to reducing the burden of HPV-associated cancer may fall short in rural America. The lack of consistent patterns of HPV-associated cancer incidence across sex, race/ethnicity, geography, and poverty warrants developing tailored, context-specific cancer prevention and control strategies to improve outcomes and advance equity in rural America.

Although much of the evidence base has focused on rural-urban comparisons, recent research has demonstrated how rural disadvantage disparately reduces healthcare access by race/ethnicity [36, 37]. Our analysis further highlights intersectional disparities in incidence within rural populations, not just by race/ethnicity, but by sex and across measures of socioeconomic status and geographic regions. Cancer risk is multifactorial. So, we expect that the observed differences across each rural subpopulation are driven by differences across a multitude of factors. While a comprehensive, intersectional analysis of preventative care and behavior in rural communities could improve our understanding, the evidence clearly shows the potential for specific risk factors for HPV-associated cancer to vary widely within rural communities.

Looking ahead, the unequal adherence to early HPV vaccination recommendations risks exacerbating the differences in rural HPV-associated cancer we observed in the 2010s. In 2010, overall HPV vaccine adherence rates were lower in males than females, AIAN and non-Hispanic Black populations than non-Hispanic White and Asian populations, and families living in poverty than more affluent families [38]. Not only did HPV vaccination adherence rates lag in southeastern states compared to coastal states, but there was significant heterogeneity within rural communities – lower adherence to HPV vaccine schedule in isolated rural towns compared to smaller, micropolitan rural towns [11, 38]. In addition to differences in historic vaccine adherence, our intersectional differences in HPV-associated cancer could also be driven by differences in high-risk HPV prevalence throughout rural southeastern communities due to riskier sexual behavior among teens [39]. Unfortunately, limited data exists quantifying the rural prevalence with population-based data. Yet, examining prevalence overall reveals startling disparities. Despite just a prevalence of 4% of the population having high-risk HPV, the prevalence ranged from 1.9% in API females to 15.8% in non-Hispanic Black males [40]. Finally, despite HPV being a primary risk factor in these HPV-associated cancers, other contributing risks may be elevated in rural communities. Consider smoking and tobacco use, both of which are more prevalent in specific rural communities as related to sex, race/ethnicity, and geography [41, 42]. Although research has yet to show the quantifiable magnitude or dose-response relationship, smoking and a high-risk HPV infection synergistically increase the risk of developing HPV-associated cancer [43, 44]. Viewing our current results in the context of these additive risks and existing evidence on the heterogeneity of tobacco-associated cancers in rural communities, efforts to reduce smoking and tobacco use may be ideal targets for reducing the burden of HPV-associated cancer in rural males, AIAN populations, and residents of the southeastern states [5].

The principles of rural health equity underscore the necessity of addressing systemic inequities by tailoring interventions to specific community contexts [45]. Community-centered approaches that leverage local strengths while addressing cultural, economic, and institutional barriers are essential to reducing HPV-associated cancer incidence among rural populations. Successful HPV vaccination interventions in rural areas must incorporate multilevel frameworks by engaging providers, community champions, and system-level leaders [46]. Given the diversity of elevated HPV-associated cancer across sex, race/ethnicity, and geography suggests that culturally adaptation will be necessary for scaling successful interventions [47]. Culturally adapted programs will integrate community-led, culturally informed strategies which improve education, preventative behavior, and access to healthcare [48, 49]. For example, combining HPV vaccination initiatives with cross-sectoral efforts in education, economic development, and health services could alleviate some of the structural disadvantages faced by especially vulnerable rural AIAN and non-Hispanic Black populations. Such strategies align with calls to expand public health planning to incorporate broader determinants of health, including economic and social factors that contribute to health outcomes.

Recognizing the systemic nature of rural health disparities, including those associated with HPV-related cancers, underscores the importance of robust surveillance systems and equitable public health infrastructure. Effective interventions must acknowledge the intersectionality of rurality, poverty, and race/ethnicity and respond with targeted efforts to alleviate the disproportionate burdens on rural minority populations. By prioritizing community-driven solutions, policymakers and practitioners can ensure that cancer prevention and control initiatives are not only equitable but also sustainable.

### Limitations

This study is not without its limitations. First, our intersectional approach stratified populations into sex and racial/ethnic categories. To minimize data suppression in groups with small cases, we aggregated data over a ten-year period. This approach prohibited us from assessing variation over time. Future research should explore alternative approaches to assess whether our findings were consistent over time. Future research could also improve upon our work, which focused on pre-2020 incidence, by exploring how intersectional differences in rural HPV-associated cancer changed before, during, and after the COVID-19 pandemic. Our results were also limited by the extent to which HPV was associated with cancer, rather than causally linked to cancer. Although the proportion of cancers causally linked to HPV, among all potentially HPV-associated cancers, varies by site, this limitation was minor as we followed established practice following CDC definitions consistently used by the existing empirical evidence in the published literature [50]. Despite aggregating over a ten-year period, some rates were still suppressed due to case counts under 15. Such suppression not only hindered our ability to comprehensively disaggregate HPV-associated cancer incidence rates by clinically relevant factors (i.e., early vs. late-stage cervical cancer), but prohibited us from even identifying sex-specific rates in certain groups. This was especially problematic for API populations, which account for less than 1% of the rural population. Further, the AIAN populations in our analysis only include residents of Purchased/Referred Care Delivery Areas – defined as counties that include at least part of an indigenous reservation or share a boundary with an indigenous reservation. The extent to which this data limitation excluded rural AIAN residents varies by state and has not been empirically quantified [51]. We also relied on sex and race/ethnicity data collected by the cancer registries, which were originally derived by medical records, patient self-reports, or cancer registry algorithms. Regarding the timing of the incidence data, the 2010–2019 data may mask rural differences in changing HPV-associated cancer rates for younger age groups driven by HPV vaccination patterns. As the initial cohorts of young people who were initially recommended to complete the HPV vaccination series in 2006 become young adults, future research can assess how introducing the HPV vaccine has impacted HPV-associated cancer rates across rural America.

## Conclusions

The findings of this study emphasize the critical need to address the complex and intersecting disparities in HPV-associated cancer incidence within rural U.S. populations. By examining such intersections of sex, race/ethnicity, socioeconomic status, and geographic region, this research highlights the need for targeted interventions and policy implementation. Addressing these disparities will require a coordinated effort that combines enhanced HPV vaccination and cervical screening programs, socioeconomic support, and culturally competent healthcare services to create equitable health outcomes for everyone living in rural communities across the U.S. We argue that these results suggest that there are few, if any, universal commonalities consistent across sex, race/ethnicity, geographic region, and poverty rates. We hope our intersectional discoveries can inform targeted strategies to reduce the burden of HPV-associated cancer across all rural communities.

## Supplementary Information

Supplementary Material 1.

Supplementary Material 2.

## Data Availability

All data for this study is reported in the supplemental exhibit 2.
