# Supplementary material for: Evaluating intersectional variation of HPV-associated cancers in rural America
Source: BMC Public Health. 2025 Aug 2;25:2627. doi: 10.1186/s12889-025-23963-y (PMC12317534; doi:10.1186/s12889-025-23963-y)
Supplement: Supplementary file 1 — Supplementary Material 1. [file 12889_2025_23963_MOESM1_ESM.docx]

**Supplemental Exhibit 1a: Selection Criteria (SEER*Stat)**

| **Site & Histology Codes** |
| --- |
| {Site and Morphology.Diagnostic Confirmation} = 'Microscopically confirmed' |
| AND {Site and Morphology.Primary Site} = 'C01.9-Base of tongue, NOS','C02.4-Lingual tonsil','C02.8-Overlapping lesion of tongue','C05.1-Soft palate, NOS','C05.2-Uvula','C09.0-Tonsillar fossa','C09.1-Tonsillar pillar','C09.8-Overlapping lesion of tonsil','C09.9-Tonsil, NOS','C10.0-Vallecula','C10.1-Anterior surface of epiglottis','C10.2-Lateral wall of oropharynx','C10.3-Posterior wall of oropharynx','C10.4-Branchial cleft','C10.8-Overlapping lesion of oropharynx','C10.9-Oropharynx, NOS','C14.0-Pharynx, NOS','C14.2-Waldeyers ring' |
| AND {Site and Morphology.Histology} = '8050/3: Papillary carcinoma, NOS','8051/3: Verrucous carcinoma, NOS','8052/3: Papillary squamous cell carcinoma','8053/3: Papillary squamous cell carcinoma, inverted','8054/3','8060/3','8070/3: Squamous cell carcinoma, NOS','8071/3: Squamous cell carcinoma, keratinizing, NOS','8072/3: Squamous cell ca., large cell, nonkeratinizing','8073/3: Squamous cell ca., small cell, nonkeratinizing','8074/3: Squamous cell carcinoma, spindle cell','8075/3: Squamous cell carcinoma, adenoid','8076/3: Squamous cell carcinoma, micro-invasive','8077/3: Squamous cell ca. & Grade III','8078/3: Squamous cell carcinoma with horn formation','8080/3: Queyrat erythroplasia, malignant','8081/3: Bowen disease, malignant','8082/3: Lymphoepithelial carcinoma','8083/3: Basaloid squamous cell carcinoma','8084/3: Squamous cell carcinoma, clear cell type','8085/3','8086/3','8121/3: Schneiderian carcinoma','8122/3: Transitional cell carcinoma, spindle cell','8123/3: Basaloid carcinoma','8124/3: Cloacogenic carcinoma','8130/3: Papillary transitional cell carcinoma','8131/3: Transitional cell carcinoma, micropapillary' |
| OR {Site and Morphology.Diagnostic Confirmation} = 'Microscopically confirmed' |
| AND {Site and Morphology.Primary Site} = 'C20.9-Rectum, NOS','C21.0-Anus, NOS','C21.1-Anal canal','C21.2-Cloacogenic zone','C21.8-Overlapping lesion of rectum, anus, and anal canal' |
| AND {Site and Morphology.Histology} = '8050/3: Papillary carcinoma, NOS','8051/3: Verrucous carcinoma, NOS','8052/3: Papillary squamous cell carcinoma','8053/3: Papillary squamous cell carcinoma, inverted','8054/3','8060/3','8070/3: Squamous cell carcinoma, NOS','8071/3: Squamous cell carcinoma, keratinizing, NOS','8072/3: Squamous cell ca., large cell, nonkeratinizing','8073/3: Squamous cell ca., small cell, nonkeratinizing','8074/3: Squamous cell carcinoma, spindle cell','8075/3: Squamous cell carcinoma, adenoid','8076/3: Squamous cell carcinoma, micro-invasive','8077/3: Squamous cell ca. & Grade III','8078/3: Squamous cell carcinoma with horn formation','8080/3: Queyrat erythroplasia, malignant','8081/3: Bowen disease, malignant','8082/3: Lymphoepithelial carcinoma','8083/3: Basaloid squamous cell carcinoma','8084/3: Squamous cell carcinoma, clear cell type','8120/3: Transitional cell carcinoma, NOS','8121/3: Schneiderian carcinoma','8122/3: Transitional cell carcinoma, spindle cell','8123/3: Basaloid carcinoma','8124/3: Cloacogenic carcinoma','8130/3: Papillary transitional cell carcinoma','8131/3: Transitional cell carcinoma, micropapillary' |
| OR {Site and Morphology.Diagnostic Confirmation} = 'Microscopically confirmed' |
| AND {Race, Sex, Year Dx, State/Province, Country.Sex} = ' Female' |
| AND {Site and Morphology.Primary Site} = 'C52.9-Vagina, NOS' |
| AND {Site and Morphology.Histology} = '8050/3: Papillary carcinoma, NOS','8051/3: Verrucous carcinoma, NOS','8052/3: Papillary squamous cell carcinoma','8053/3: Papillary squamous cell carcinoma, inverted','8054/3','8060/3','8070/3: Squamous cell carcinoma, NOS','8071/3: Squamous cell carcinoma, keratinizing, NOS','8072/3: Squamous cell ca., large cell, nonkeratinizing','8073/3: Squamous cell ca., small cell, nonkeratinizing','8074/3: Squamous cell carcinoma, spindle cell','8075/3: Squamous cell carcinoma, adenoid','8076/3: Squamous cell carcinoma, micro-invasive','8077/3: Squamous cell ca. & Grade III','8078/3: Squamous cell carcinoma with horn formation','8080/3: Queyrat erythroplasia, malignant','8081/3: Bowen disease, malignant','8082/3: Lymphoepithelial carcinoma','8083/3: Basaloid squamous cell carcinoma','8084/3: Squamous cell carcinoma, clear cell type','8120/3: Transitional cell carcinoma, NOS','8121/3: Schneiderian carcinoma','8122/3: Transitional cell carcinoma, spindle cell','8123/3: Basaloid carcinoma','8124/3: Cloacogenic carcinoma','8130/3: Papillary transitional cell carcinoma','8131/3: Transitional cell carcinoma, micropapillary','8153/3: Gastrinoma, malignant' |
| OR {Site and Morphology.Diagnostic Confirmation} = 'Microscopically confirmed' |
| AND {Race, Sex, Year Dx, State/Province, Country.Sex} = ' Male' |
| AND {Site and Morphology.Primary Site} = 'C60.9-Penis, NOS' |
| AND {Site and Morphology.Histology} = '8054/3','8060/3','8070/3: Squamous cell carcinoma, NOS','8071/3: Squamous cell carcinoma, keratinizing, NOS','8072/3: Squamous cell ca., large cell, nonkeratinizing','8073/3: Squamous cell ca., small cell, nonkeratinizing','8074/3: Squamous cell carcinoma, spindle cell','8075/3: Squamous cell carcinoma, adenoid','8076/3: Squamous cell carcinoma, micro-invasive','8077/3: Squamous cell ca. & Grade III','8078/3: Squamous cell carcinoma with horn formation','8080/3: Queyrat erythroplasia, malignant','8081/3: Bowen disease, malignant','8082/3: Lymphoepithelial carcinoma','8083/3: Basaloid squamous cell carcinoma','8084/3: Squamous cell carcinoma, clear cell type','8120/3: Transitional cell carcinoma, NOS','8121/3: Schneiderian carcinoma','8122/3: Transitional cell carcinoma, spindle cell','8123/3: Basaloid carcinoma','8124/3: Cloacogenic carcinoma','8130/3: Papillary transitional cell carcinoma','8131/3: Transitional cell carcinoma, micropapillary' |
| OR {Site and Morphology.Diagnostic Confirmation} = 'Microscopically confirmed' |
| AND {Race, Sex, Year Dx, State/Province, Country.Sex} = ' Female' |
| AND {Site and Morphology.Primary Site} = 'C53.0-Endocervix','C53.1-Exocervix','C53.8-Overlapping lesion of cervix uteri','C53.9-Cervix uteri' |
| AND ({Site and Morphology.Histology} = '8000/3: Neoplasm, malignant','8001/3: Tumor cells, malignant','8002/3: Malignant tumor, small cell type','8003/3: Malignant tumor, giant cell type','8004/3: Malignant tumor, spindle cell type','8005/3: Malignant tumor, clear cell type','8010/3: Carcinoma, NOS','8011/3: Epithelioma, malignant','8012/3: Large cell carcinoma, NOS','8013/3: Large cell neuroendocrine carcinoma','8014/3: Large cell carcinoma with rhabdoid phenotype','8015/3: Glassy cell carcinoma','8020/3: Carcinoma, undifferentiated type, NOS','8021/3: Carcinoma, anaplastic type, NOS','8022/3: Pleomorphic carcinoma','8023/3','8030/3: Giant cell and spindle cell carcinoma','8031/3: Giant cell carcinoma','8032/3: Spindle cell carcinoma','8033/3: Pseudosarcomatous carcinoma','8034/3: Polygonal cell carcinoma','8035/3: Carcinoma with osteoclast-like giant cells','8040/3: Tumorlet, malignant','8041/3: Small cell carcinoma, NOS','8042/3: Oat cell carcinoma','8043/3: Small cell carcinoma, fusiform cell','8044/3: Small cell carcinoma, intermediate cell','8045/3: Combined small cell carcinoma','8046/3: Non-small cell carcinoma','8050/3: Papillary carcinoma, NOS','8051/3: Verrucous carcinoma, NOS','8052/3: Papillary squamous cell carcinoma','8053/3: Papillary squamous cell carcinoma, inverted','8054/3','8060/3','8070/3: Squamous cell carcinoma, NOS','8071/3: Squamous cell carcinoma, keratinizing, NOS','8072/3: Squamous cell ca., large cell, nonkeratinizing','8073/3: Squamous cell ca., small cell, nonkeratinizing','8074/3: Squamous cell carcinoma, spindle cell','8075/3: Squamous cell carcinoma, adenoid','8076/3: Squamous cell carcinoma, micro-invasive','8077/3: Squamous cell ca. & Grade III','8078/3: Squamous cell carcinoma with horn formation','8080/3: Queyrat erythroplasia, malignant','8081/3: Bowen disease, malignant','8082/3: Lymphoepithelial carcinoma','8083/3: Basaloid squamous cell carcinoma','8084/3: Squamous cell carcinoma, clear cell type','8085/3','8086/3','8090/3: Basal cell carcinoma, NOS','8091/3: Multifocal superficial basal cell carcinoma','8092/3: Infiltrating basal cell carcinoma, NOS','8093/3: Basal cell carcinoma, fibroepithelial','8094/3: Basosquamous carcinoma','8095/3: Metatypical carcinoma','8097/3: Basal cell carcinoma, nodular','8098/3: Adenoid basal cell carcinoma','8100/3: Trichoepithelioma, malignant','8101/3','8102/3: Trichilemmocarcinoma','8103/3: Pilar tumor, malignant','8110/3: Pilomatrix carcinoma','8120/3: Transitional cell carcinoma, NOS','8121/3: Schneiderian carcinoma','8122/3: Transitional cell carcinoma, spindle cell','8123/3: Basaloid carcinoma','8124/3: Cloacogenic carcinoma','8130/3: Papillary transitional cell carcinoma','8131/3: Transitional cell carcinoma, micropapillary','8140/3: Adenocarcinoma, NOS','8141/3: Scirrhous adenocarcinoma','8142/3: Linitis plastica','8143/3: Superficial spreading adenocarcinoma','8144/3: Adenocarcinoma, intestinal type','8145/3: Carcinoma, diffuse type','8146/3: Monomorphic adenocarcinoma','8147/3: Basal cell adenocarcinoma','8148/3: Glandular neoplasia, malignant, Grade III','8149/3','8150/3: Islet cell carcinoma','8151/3: Insulinoma, malignant','8152/3: Glucagonoma, malignant','8153/3: Gastrinoma, malignant','8154/3: Mixed islet cell and exocrine adenocarcinoma','8155/3: Vipoma','8156/3: Somatostatinoma, malignant','8158/3','8160/3: Cholangiocarcinoma','8161/3: Bile duct cystadenocarcinoma','8162/3: Klatskin tumor','8163/3','8170/3: Hepatocellular carcinoma, NOS','8171/3: Hepatocellular carcinoma, fibrolamellar','8172/3: Hepatocellular carcinoma, scirrhous','8173/3: Hepatocellular carcinoma, spindle cell variant','8174/3: Hepatocellular carcinoma, clear cell type','8175/3: Hepatocellular carcinoma, pleomorphic type','8180/3: Combined hepatocellular ca. & cholangiocarcinoma','8190/3: Trabecular adenocarcinoma','8200/3: Adenoid cystic carcinoma','8201/3: Cribriform carcinoma','8210/3: Adenocarcinoma in adenomatous polyp','8211/3: Tubular adenocarcinoma','8212/3','8213/3: Serrated adenocarcinoma','8214/3: Parietal cell carcinoma','8215/3: Adenocarcinoma of anal glands','8220/3: Adenocarcinoma in adenomatous polyposis coli','8221/3: Adenocarcinoma in mult. adenomatous polypsosis coli','8230/3: Solid carcinoma, NOS','8231/3: Carcinoma simplex','8240/3: Carcinoid tumor, malignant','8241/3: Enterochromaffin cell carcinoid','8242/3: Enterochromaffin-like cell tumor, malignant','8243/3: Goblet cell carcinoid','8244/3: Composite carcinoid','8245/3: Adenocarcinoid tumor','8246/3: Neuroendocrine carcinoma','8247/3: Merkel cell carcinoma','8248/3: Apudoma','8249/3: Atypical carcinoid tumor','8250/3: Bronchiolo-alveolar adenocarcinoma','8251/3: Alveolar adenocarcinoma','8252/3: Bronchiolo-alveolar carcinoma, non-mucinous','8253/3: Bronchiolo-alveolar carcinoma, mucinous','8254/3: Bronchiolo-alveolar ca., mucinous & non-mucinous','8255/3: Adenocarcinoma with mixed subtypes','8256/3','8257/3','8260/3: Papillary adenocarcinoma, NOS','8261/3: Adenocarcinoma in villous adenoma','8262/3: Villous adenocarcinoma','8263/3: Adenocarcinoma in tubulovillous adenoma','8265/3','8270/3: Chromophobe carcinoma','8271/3: Prolactinoma, malignant','8272/3: Pituitary carcinoma, NOS','8280/3: Acidophil carcinoma','8290/3: Oxyphilic adenocarcinoma','8310/3: Clear cell adenocarcinoma, NOS','8311/3','8312/3: Renal cell carcinoma','8313/3: Clear cell adenocarcinofibroma','8314/3: Lipid-rich carcinoma','8315/3: Glycogen-rich carcinoma','8316/3: Cyst-associated renal cell carcinoma','8317/3: Renal cell carcinoma, chromophobe type','8318/3: Renal cell carcinoma, sarcomatoid','8319/3: Collecting duct carcinoma','8320/3: Granular cell carcinoma','8322/3: Water-clear cell adenocarcinoma','8323/3: Mixed cell adenocarcinoma','8330/3: Follicular adenocarcinoma, NOS','8331/3: Follicular adenocarcinoma well differentiated','8332/3: Follicular adenocarcinoma trabecular','8333/3: Fetal adenocarcinoma','8335/3: Follicular carcinoma, minimally invasive','8336/3','8337/3: Insular carcinoma','8339/3','8340/3: Papillary carcinoma, follicular variant','8341/3: Papillary microcarcinoma','8342/3: Papillary carcinoma, oxyphilic cell','8343/3: Papillary carcinoma, encapsulated','8344/3: Papillary carcinoma, columnar cell','8345/3: Medullary carcinoma with amyloid stroma','8346/3: Mixed medullary-follicular carcinoma','8347/3: Mixed medullary-papillary carcinoma','8350/3: Nonencapsulated sclerosing carcinoma','8360/3','8361/3','8370/3: Adrenal cortical carcinoma','8373/3','8380/3: Endometrioid carcinoma','8381/3: Endometrioid adenofibroma, malignant','8382/3: Endometrioid adenocarcinoma, secretory variant','8383/3: Endometrioid adenocarcinoma, ciliated cell variant','8384/3: Adenocarcinoma, endocervical type','8390/3: Skin appendage carcinoma','8400/3: Sweat gland adenocarcinoma','8401/3: Apocrine adenocarcinoma','8402/3: Nodular hidradenoma, malignant','8403/3: Malignant eccrine spiradenoma','8406/3: Papillary syringadenocarcinoma','8407/3: Sclerosing sweat duct carcinoma','8408/3: Eccrine papillary adenocarcinoma','8409/3: Eccrine poroma, malignant','8410/3: Sebaceous adenocarcinoma','8413/3: Eccrine adenocarcinoma','8420/3: Ceruminous adenocarcinoma','8430/3: Mucoepidermoid carcinoma','8440/3: Cystadenocarcinoma, NOS','8441/3: Serous cystadenocarcinoma, NOS','8442/3: Proliferating serous carcinoma','8443/3: Clear cell cystadenocarcinoma','8444/3: Clear cell cystic tumor, malignant','8450/3: Papillary cystadenocarcinoma, NOS','8452/3: Solid pseudopapillary carcinoma','8453/3: Intraductal papillary-mucinous carcinoma, invasive','8460/3: Papillary serous cystadenocarcinoma','8461/3: Serous surface papillary carcinoma','8462/3: Papillary serous cystadenocarcinoma','8463/3: Serous surface papillary carcinoma','8470/3: Mucinous cystadenocarcinoma, NOS','8471/3: Papillary mucinous cystadenocarcinoma','8472/3: Mucinous cystadenocarcinoma','8473/3: Papillary pseudomucinous cystadenocarcinoma','8474/3','8480/3: Mucinous adenocarcinoma','8481/3: Mucin-producing adenocarcinoma','8482/3: Mucinous adenocarcinoma, endocervical type','8490/3: Signet ring cell carcinoma','8500/3: Infiltrating duct carcinoma, NOS','8501/3: Comedocarcinoma, NOS' |
| OR {Site and Morphology.Histology} = '8502/3: Secretory carcinoma of breast','8503/3: Intraductal papillary adenocarcinoma with invasion','8504/3: Intracystic carcinoma, NOS','8507/3: Ductal carcinoma, micropapillary','8508/3: Cystic hypersecretory carcinoma','8509/3','8510/3: Medullary carcinoma, NOS','8512/3: Medullary carcinoma with lymphoid stroma','8513/3: Atypical medullary carcinoma','8514/3: Duct carcinoma, desmoplastic type','8520/3: Lobular carcinoma, NOS','8521/3: Infiltrating ductular carcinoma','8522/3: Infiltrating duct and lobular carcinoma','8523/3: Infiltrating duct mixed with other types of ca.','8524/3: Infiltrating lobular mixed with other types of ca.','8525/3: Polymorphous low grade adenocarcinoma','8530/3: Inflammatory carcinoma','8540/3: Paget disease, mammary','8541/3: Paget disease and infiltrating duct carcinoma','8542/3: Paget disease, extramammary','8543/3: Paget disease and intraductal carcinoma','8550/3: Acinar cell carcinoma','8551/3: Acinar cell cystadenocarcinoma','8552/3','8560/3: Adenosquamous carcinoma','8561/3: Warthin tumor, malignant','8562/3: Epithelial-myoepithelial carcinoma','8570/3: Adenocarcinoma with squamous metaplasia','8571/3: Adenocarcinoma w. cartilaginous & osseous metaplasia','8572/3: Adenocarcinoma with spindle cell metaplasia','8573/3: Adenocarcinoma with apocrine metaplasia','8574/3: Adenocarcinoma with neuroendocrine differentiation','8575/3: Metaplastic carcinoma, NOS','8576/3: Hepatoid adenocarcinoma','8580/3: Thymoma, malignant, NOS','8581/3: Thymoma, type A, malignant','8582/3: Thymoma, type AB, malignant','8583/3: Thymoma, type B1, malignant','8584/3: Thymoma, type B2, malignant','8585/3: Thymoma, type B3, malignant','8586/3: Thymic carcinoma, NOS','8588/3: Spindle epithelial tumor with thymus-like element','8589/3: Carcinoma showing thymus-like element','8590/3: Sex cord-gonadal stromal tumor, malignant, NOS','8591/3','8592/3','8593/3: Malignant Stromal tumor, with sex cord elements','8600/3: Thecoma, malignant','8610/3','8620/3: Granulosa cell tumor, malignant','8621/3: Granulosa cell-theca cell tumor, malignant','8622/3: Juvenile granulosa cell tumor, malignant','8623/3: Sex cord tumor with annular tubules, malignant','8630/3: Androblastoma, malignant','8631/3: Sertoli-Leydig cell tumor, poorly differentiated','8632/3: Gynandroblastoma, malignant','8634/3: Sertoli-Leydig, poor diff. w. heterologous elements','8640/3: Sertoli cell carcinoma','8642/3','8650/3: Leydig cell tumor, malignant','8670/3: Steroid cell tumor, malignant','8940/3: Mixed tumor, malignant, NOS','8941/3: Carcinoma in pleomorphic adenoma') |
|  |
| **Race/Rural Codes** |
| {Race and Ethnicity.Race and Ethnicity recode} = 'Hispanic, All Races',' Non-Hispanic White',' Non-Hispanic Black',' Non-Hispanic API',' Non-Hispanic AIAN PRCDA' |
| AND {County Level ABSMs.Metro/Non-Metro (2013 Beale)} = 'Non-Metro' |
|  |
| **Year/Country Codes** |
| AND {Sex, Year Dx, State/Province, Country.Year of diagnosis} = '2010','2011','2012','2013','2014','2015','2016','2017','2018','2019' |
| AND {Sex, Year Dx, State/Province, Country.Country} = ' United States' |

**Regional Breakdown:**

We defined these regions as:

Northeast (Connecticut, Delaware, Maine, Maryland, Massachusetts, New Hampshire, New Jersey, New York, Pennsylvania, Rhode Island, Vermont, Washington, D.C.);

Midwest (Illinois, Indiana, Iowa, Kansas, Michigan, Minnesota, Missouri, Nebraska, North Dakota, Ohio, South Dakota, Wisconsin);

Southeast (Alabama, Arkansas, Florida, Georgia, Kentucky, Louisiana, Mississippi, North Carolina, Oklahoma, South Carolina, Tennessee, Texas, Virginia, West Virginia);

West (Alaska, Arizona, California, Colorado, Hawaii, Idaho, Montana, Nevada, New Mexico, Oregon, Utah, Washington, Wyoming).

**Supplemental Exhibit 1b - HPV-associated Cancer Cases with Missing Race/Ethnicity Data**

|  | **Male** | **Female** |
| --- | --- | --- |
| **Overall** | 196 | 298 |
| **Age** |  |  |
| 0-29 | <16 | 16 |
| 30-49 | 27 | 122 |
| 50-64 | 102 | 103 |
| 65-79 | 58 | 50 |
| 80+ | <16 | <16 |
|  |  |  |
| **Poverty** |  |  |
| <10% | 35 | 48 |
| 10-20% | 82 | 94 |
| >20% | 60 | 117 |
|  |  |  |
| **Region** |  |  |
| Northeast | <16 | <16 |
| Midwest | 64 | 89 |
| Southeast | 76 | 138 |
| West | 43 | 57 |

Supplemental Exhibit 1b reports the number of HPV-associated cancer cases in rural communities (RUCC 4-9), diagnosed between 2010-2019, with missing race/ethnicity data. Cases with counts less than 16 were suppressed.
